# Neutrophil-to-lymphocyte ratio is associated with early cervical shortening after cerclage: a single-center cohort study integrating C-reactive protein

**DOI:** 10.3389/fmed.2026.1843992

**Published:** 2026-06-18

**Authors:** Yuance Xu, Linlin Zhang, Shuang Guo, Guochao Jiang, Zheyu Song, Jia Song, Zongxiang Leng, Xingwei Zhang

**Affiliations:** Department of Gynecology, Jilin Women And Children Health Hospital (Jilin Province Maternal and Child Health Quality Control Center), Changchun, China

**Keywords:** atosiban, cervical cerclage, C-reactive protein, neutrophil-to-lymphocyte ratio, preterm birth

## Abstract

**Objective:**

This study aimed to explore whether pre-operative systemic inflammatory markers—neutrophil-to-lymphocyte ratio (NLR) and C-reactive protein (CRP)—are associated with early post-cerclage cervical shortening and to examine whether atosiban use varied according to inflammatory status in this retrospective cohort.

**Methods:**

A single-center retrospective cohort study conducted in 2023 included 84 singleton pregnancies that underwent McDonald cerclage. Full blood count and CRP were measured within 24 h before surgery. The primary outcomes were (i) the cervical length (CL) shortening rate (mm/week) up to 28 weeks’ gestation and (ii) spontaneous preterm birth before 32 weeks’ gestation. Atosiban was administered at the attending physician’s discretion to women with threatened preterm labor or recurrent uterine contractions, not by random allocation. Treatment allocation was non-random and susceptible to confounding by indication.

**Results:**

NLR correlated linearly with CL shortening (*r* = 0.61, *p* < 0.001). A multivariable regression analysis showed that each 1-unit increase in NLR was associated with an additional 0.09 mm/week of cervical shortening. Among high-NLR (≥3.5) women, PTB before 32 weeks’ gestation occurred in 60.0% (3/5) of those without atosiban compared with 14.3% (3/21) of those receiving atosiban, compared with 0% (0/58) in women with low NLR (<3.5). Prolonged antibiotics lowered post-operative CRP but did not alter CL loss. Atosiban exposure was differentially distributed by NLR status; however, due to confounding by indication and critically small subgroups, no inference regarding atosiban efficacy or clinical utility can be supported. Combined NLR and CRP predicted large shortening (ΔCL ≤ −5 mm) with an area under the curve (AUC) of 0.81.

**Conclusion:**

In this small (*n* = 84), single-center, retrospective cohort, pre-operative NLR and CRP were statistically associated with accelerated cervical shortening after cerclage. Atosiban and antibiotic exposure were determined by clinical indication, and subgroup analyses were based on severely inadequate sample sizes (*n* = 5, *n* = 2) with extreme confounding by indication. These findings are exploratory observations only. No causal inference, therapeutic claim, or clinical recommendation is justified. Prospective validation in randomized trials is essential.

## Introduction

Despite the established efficacy of cervical cerclage in prolonging gestation among women with cervical insufficiency, 15–30% of treated pregnancies still culminate in spontaneous preterm birth before 32 weeks’ gestation (1, 2). This residual failure rate suggests the presence of underlying pathological processes that mechanical reinforcement alone cannot overcome. Accumulating histopathological and translational evidence indicates that subclinical intrauterine inflammation may contribute to cervical stromal remodeling long before clinical signs emerge (3, 4). Identifying an objective, readily accessible marker of this inflammatory milieu remains a critical translational gap.

The peripheral blood immunogram offers an under-exploited window into this covert process. The neutrophil-to-lymphocyte ratio (NLR)—a dimensionless derivative of the routine full blood count—encodes the balance between innate neutrophil activation and adaptive lymphocyte regulation, and can increase within hours of a cytokine surge (5). C-reactive protein (CRP), synthesized hepatically in response to interleukin-6 signaling, provides a complementary readout of systemic acute-phase intensity (6, 7). Critically, both biomarkers increase after surgical trauma such as cerclage insertion; however, their pre-operative levels have not been interrogated as quantitative predictors of subsequent cervical shortening velocity. Specifically, an NLR threshold of ≥3.5 may identify women at the highest risk for inflammation-driven cervical remodeling (8, 9).

In addition to prediction, an equally fundamental question is whether this process is modifiable. Classical antibiotics can sterilize bacteremia but leave aseptic, cytokine-driven collagenolysis intact (10, 11). Conversely, the oxytocin receptor antagonist atosiban has been shown in *ex vivo* human myometrial models to inhibit nuclear factor kappa-light-chain-enhancer of activated B cells (NF-κB) and mitogen-activated protein kinase (MAPK) activation and to reduce cyclooxygenase-2 (COX-2) expression—experimental observations distinct from its established tocolytic role (12, 13). Whether these *in vitro* pharmacological properties translate into measurable effects on cervical remodeling *in vivo* remains unknown and has not been tested in controlled clinical trials.

We therefore designed a single-center retrospective cohort study to interrogate two intertwined hypotheses: First, pre-operative NLR and CRP operate as continuous, dose-dependent signals of post-cerclage cervical length loss, and second, atosiban use may differ according to baseline inflammatory status, with potential implications for cervical remodeling. Demonstrating these associations could support further investigation into whether routine blood parameters might inform risk stratification and individualized management after cervical cerclage.

## Materials and methods

### Study design and ethical oversight

A single-center retrospective cohort study conducted between January 2024 and December 2025 at Jilin Provincial Maternal and Child Health Hospital examined whether pre-operative inflammatory indices are associated with post-cerclage cervical remodeling and whether atosiban exposure differs by inflammatory status. Institutional Review Board approval (No. 20220011) was obtained.

### Participants

Consecutive singleton pregnancies (12–26 weeks) that received McDonald cerclage were screened. The inclusion criteria were as follows: (i) history- or ultrasound-indicated cerclage; (ii) full blood count and serum CRP collected ≤ 24 h before surgery; and (iii) at least two transvaginal cervical length (CL) measurements up to 28 weeks. The exclusion criteria were as follows: multiple pregnancy, ruptured membranes, chorioamnionitis, active infection (temperature ≥ 38 °C or CRP ≥ 20 mg/L), hematological disorders, or incomplete blood data. A total of 84 women constituted the final analytic sample, comprising 26 high-NLR (≥3.5) and 58 low-NLR (<3.5) women.

### Inflammatory exposure variables

Neutrophil, lymphocyte, and monocyte counts were extracted from the pre-operative full blood count; NLR was computed as the neutrophil count divided by the lymphocyte count. CRP was analyzed both continuously and dichotomized at 5 mg/L, a threshold commonly used in clinical practice to distinguish normal from mildly elevated systemic inflammation. In addition, the C-reactive protein level was also recorded on the second day after the operation to verify the systemic inflammatory response.

### Pharmacological exposures and confounding by indication

Treatment allocation was non-random and determined by clinical indication. Total exposure days to two pharmacological regimens were quantified from electronic prescriptions:

1. Intravenous antibiotics—any *β*-lactam or macrolide—were prescribed for prophylaxis or suspected infection.
2. Atosiban (oxytocin receptor antagonist) was administered as a 6-h bolus followed by continuous infusion. It was prescribed for threatened preterm labor or recurrent uterine contractions at the attending physician’s discretion.

Since atosiban was prescribed for threatened preterm labor and antibiotics for suspected infection or prophylaxis, treatment groups differed systematically in baseline disease severity. Pre-operative NLR was 4.29 ± 1.30 in atosiban-exposed women and 2.73 ± 1.18 in atosiban-unexposed women (*p* < 0.0001); similarly, women receiving ≥4 days of antibiotics had a pre-operative NLR of 4.38 ± 1.72 compared to 2.51 ± 0.45 in those receiving <4 days (*p* < 0.0001). These analyses, therefore, compare differential exposure by clinical indication and not treatment effects. Causal interpretation is not justified.

Duration of each drug (days) was treated as both continuous and categorical (≥ 4 vs. < 4 days) to allow exploratory dose–response evaluation.

### Outcomes

The primary outcomes were:

(i) the rate of cervical length shortening (mm/week) from baseline to 28 weeks’ gestation or delivery and (ii) spontaneous preterm birth before 32 weeks’ gestation. The secondary outcomes included delivery before 35 weeks’ gestation, post-operative CRP trajectory, and composite neonatal morbidity (Respiratory Distress Syndrome (RDS), Intraventricular Hemorrhage (IVH), Necrotizing Enterocolitis (NEC), and culture-proven sepsis).

### Cervical length measurement protocol

A transvaginal ultrasound was performed by trained sonographers using a 5–7.5-MHz probe with an empty bladder. CL was measured as the linear distance between the internal and external os along the endocervical canal, visualized in the sagittal plane. Three measurements were obtained, and the shortest valid measurement was recorded. Baseline CL was measured within 24 h pre-cerclage; follow-up measurements were scheduled at 2-week intervals up to 28 weeks or delivery, whichever occurred first. The shortening rate was calculated as (final CL minus baseline CL) divided by the number of weeks elapsed.

### Statistical analysis

Normality was assessed using Shapiro–Wilk tests. Continuous data are presented as mean ± standard deviation (SD) or median (interquartile range [IQR]); categorical data are presented as n (%). The Pearson/Spearman coefficients were used to evaluate univariate correlations. A multivariable linear regression was used to estimate the independent association between NLR or CRP and CL shortening rate after adjusting for age, body mass index (BMI), previous PTB, cerclage indication, baseline CL, gestational age at cerclage, uterine contraction status at admission (documented as present or absent based on tocography), and cumulative drug days. Interaction terms (NLR × atosiban; CRP × antibiotics) were included to test modifiability. The receiver operating characteristic (ROC) curves were used to compare the predictive accuracy of NLR, CRP, and their combination for large shortening (ΔCL ≤ −5 mm). To mitigate overfitting risk, we used bootstrap resampling (1,000 iterations) to estimate confidence intervals for regression coefficients and performed sensitivity analyses with backward stepwise variable selection based on the Akaike information criterion. A two-tailed *p*-value of < 0.05 was considered significant. Analyses were performed using R 4.2.1 and SPSS 26.0.

## Results

### Association between inflammatory markers and cervical shortening

**Figure 1:**
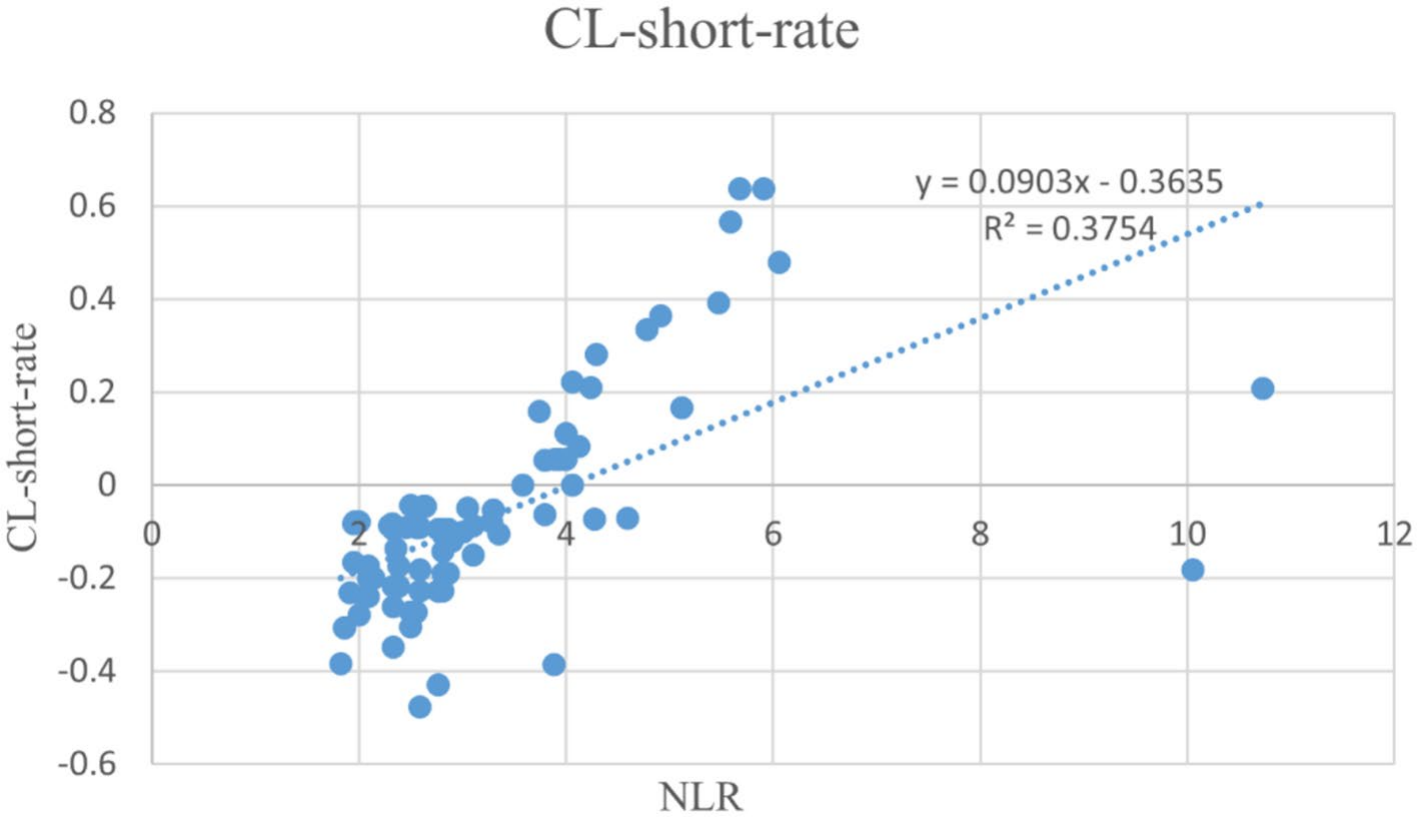
Correlation between pre-operative neutrophil-to-lymphocyte ratio (NLR) and cervical length shortening rate after cerclage. Scatter plot showing a linear relationship (*r* = 0.61, *p* < 0.001) between the NLR and the rate of cervical length change (mm/week) from baseline to 28 weeks or delivery.

The single-factor analysis showed that the neutrophil-to-lymphocyte ratio (NLR) was linearly positively correlated with the rate of cervical length (CL) shortening (*r* = 0.61, *p* < 0.001). The multivariate linear model, after adjusting for age, BMI, previous history of pre-term birth, CL at the time of surgery, and the number of medication days, indicated that, for every 1-unit increase in NLR, the additional loss of CL was 0.09 mm/week (95% CI 0.06–0.12; *p* < 0.001). Monocytes of ≥ 0.5 × 10 (9)/L were also associated with accelerated shortening (*β* = 0.04 mm/week, *p* = 0.01), suggesting that multiple inflammatory signals in the complete blood count may jointly contribute to prediction (Figure 1) (5). The clinical relevance of 0.09 mm/week is modest in absolute terms but may be significant over the 10–16-week follow-up period: a woman with an NLR of 5.0 would be expected to lose approximately 0.45 mm/week more than a woman with an NLR of 1.0, translating to an additional 4.5–7.2 mm over the observation window—a magnitude that approaches or exceeds the measurement error of transvaginal ultrasound (±2–3 mm) but lies at the lower boundary of detectability. To contextualize against measurement variability, transvaginal cervical length measurement has reported intra-observer variability of ±1.5–2.5 mm and inter-observer variability of ±2–3 mm per measurement. Over a 16-week follow-up with measurements every 2 weeks (8 measurements total), the cumulative measurement error approximates ±0.09–0.19 mm/week. The observed coefficient, therefore, lies at the lower boundary of biological detectability and should be interpreted as a statistical association of uncertain clinical actionability.

### Antibiotic exposure and cervical remodeling: no causal inference possible

Antibiotic duration was determined by clinical indication rather than randomization. Women with higher baseline inflammation received longer courses, reflecting confounding by indication. Any observed differences in cervical shortening likely reflect baseline disease severity rather than a causal effect of antibiotics. Although prolonged intravenous antibiotics for ≥4 days was administered to women with significantly higher baseline inflammation (pre-operative CRP: 6.13 ± 2.36 vs. 3.51 ± 0.65 mg/L, *p* < 0.0001; NLR: 4.38 ± 1.72 vs. 2.51 ± 0.45, *p* < 0.0001), post-operative CRP on day 2 remained elevated in the long-course group compared to the short-course group (6.46 ± 1.60 vs. 3.99 ± 1.00 mg/L, *p* < 0.0001; Table 1). Notably, the cervical length shortening rate differed significantly between the two groups (−0.18 ± 0.10 vs. 0.08 ± 0.35 mm/week, *p* < 0.0001). Women who received <4 days of antibiotics showed cervical lengthening post-cerclage (negative value indicating a mechanical support effect), whereas those who received ≥4 days showed active cervical shortening despite prolonged antimicrobial therapy.

**Table 1:** Effect of prolonged antibiotic therapy on inflammatory markers and cervical length shortening in women undergoing cervical cerclage.

Index	Antibiotics used for less than 4 days (*n* = 55)	Antibiotics used for more than 4 days (*n* = 29)	*p*-value
Pre-operation CRP (mg/L)	3.51 ± 0.65	6.13 ± 2.36	<0.0001
Pre-operation NLR	2.51 ± 0.45	4.38 ± 1.72	<0.0001
The second day after the operation, CRP (mg/L)	3.99 ± 1.00	6.46 ± 1.60	<0.0001
CL shortening rate (mm/week)	−0.18 ± 0.10	0.08 ± 0.35	<0.0001

Data are presented as mean ± standard deviation. CRP, C-reactive protein; NLR, neutrophil-to-lymphocyte ratio; CL, cervical length.

The persistent elevation of post-operative CRP, despite prolonged antibiotic coverage, is consistent with ongoing sterile intrauterine inflammation that may not be responsive to antimicrobial therapy; however, this interpretation is speculative, given the non-randomized design (10, 11).

### Atosiban exposure and cervical length: a confounded observational comparison

Atosiban was selectively prescribed to women with threatened preterm labor who had higher baseline NLR, CRP, and preterm birth history. The following comparisons are observational and confounded by indication; they should not be interpreted as estimates of atosiban efficacy. Atosiban was selectively administered to women with higher baseline inflammation (pre-operative NLR 4.29 ± 1.30 vs. 2.73 ± 1.18, *p* < 0.0001; CRP 5.80 ± 0.61 vs. 3.89 ± 2.00 mg/L, *p* < 0.0001) and a predominant history of preterm birth (82.6% vs. 0%, *p* < 0.0001). In an unadjusted analysis, high-NLR women receiving atosiban showed attenuated cervical shortening compared to their untreated counterparts (0.10 ± 0.22 vs. 0.25 ± 0.28 mm/week). In contrast, low-NLR women showed cervical lengthening regardless of atosiban exposure (−0.18 ± 0.10, n = 56 vs. −0.16 ± 0.02 mm/week, *n* = 2; Table 2).

**Table 2:** Effect of atosiban on cervical length shortening stratified by pre-operative neutrophil-to-lymphocyte ratio (NLR) status.

Hierarchy	Atosiban	n	CL shortening rate (mm/week)	*p*-value
High NLR (≥3.5)	Without atosiban	5	+0.25 ± 0.28	-
High NLR (≥3.5)	Indicates atosiban	21	+0.10 ± 0.22	0.026
Low NLR (<3.5)	Without atosiban	56	−0.18 ± 0.10	-
Low NLR (<3.5)	Indicates atosiban	2	−0.16 ± 0.02	0.65

**Figure 2:**
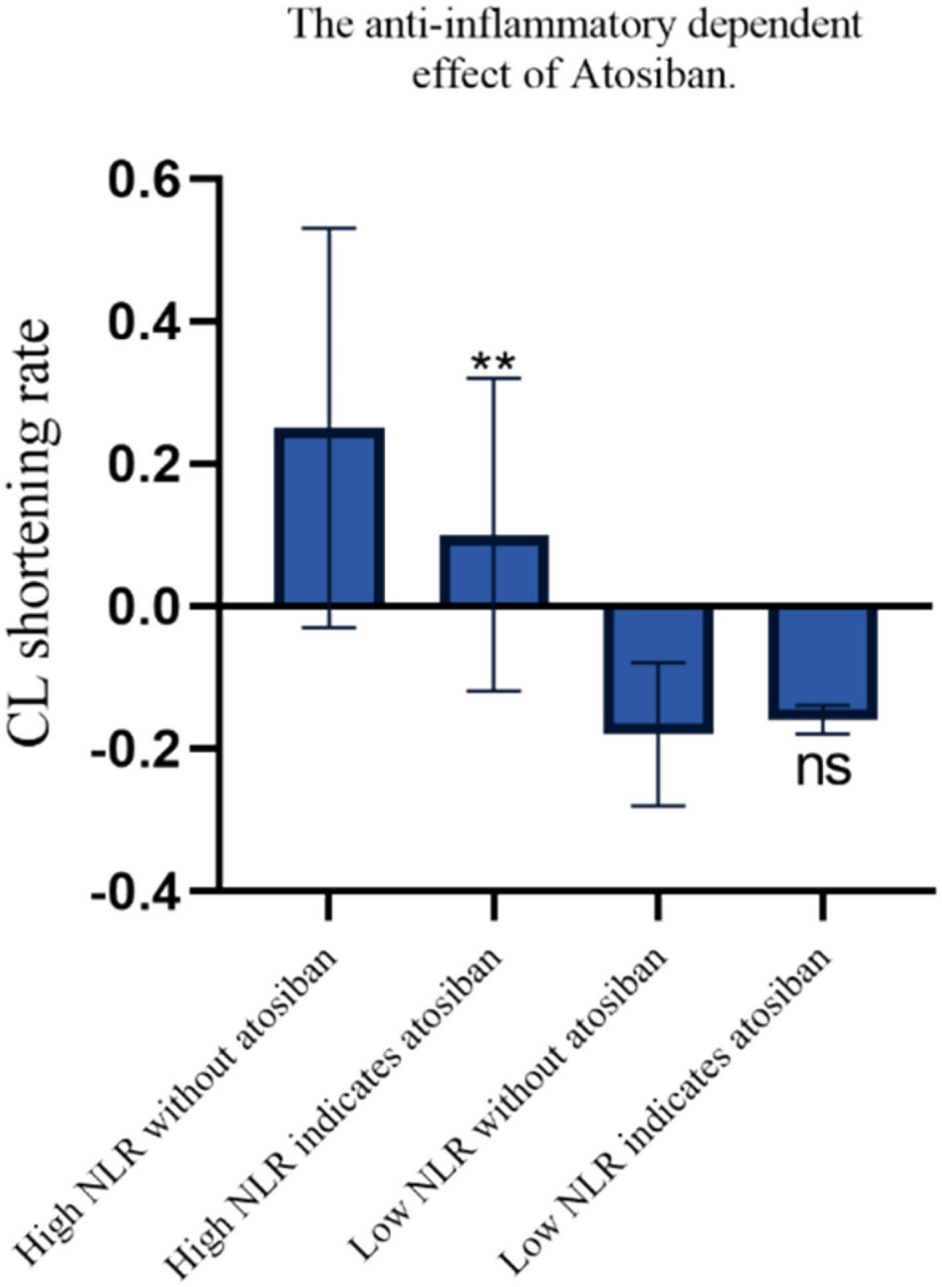
Anti-inflammatory dependent effect of atosiban on cervical length shortening. **(A)** Cervical length shortening rate in high/low NLR groups with/without atosiban; **(B)** Post-operative changes in NLR and CRP in high-NLR women receiving atosiban. Error bars represent standard deviation. *p < 0.05 for interaction.

Following the multivariable adjustment, atosiban was associated with a 0.15-mm/week lower shortening rate in the high-NLR stratum (interaction *p* = 0.026), with no apparent association in low-NLR women (adjusted difference of −0.02 mm/week, *p* = 0.65). Concurrent reductions in post-operative NLR (mean −0.6 units) and CRP were observed in high-NLR atosiban recipients (Figure 2) (12, 13). These results are exploratory and should be interpreted with substantial caution. The subgroup sizes were highly imbalanced (high-NLR women without atosiban, *n* = 5; high-NLR women with atosiban, *n* = 21), and the treatment was not randomized. The observed association may reflect confounding by indication—women prescribed atosiban may have differed in unmeasured ways from those who did not receive it. The interaction *p*-value should be viewed as hypothesis-generating rather than confirmatory. The high-NLR subgroup without atosiban comprised only five patients, and the low-NLR subgroup with atosiban comprised only two patients. The approximate 95% confidence interval for the high-NLR/atosiban-naïve mean spanned 0.25 ± 0.35 mm/week (approximately −0.10 to +0.60), indicating that the true mean could plausibly range from cervical lengthening to severe shortening. Bootstrap percentile intervals (1,000 resamples) confirmed extreme instability: The 2.5th–97.5th percentile for the high-NLR/atosiban-naïve mean was −0.02 to +0.58 mm/week. Estimates derived from the high-NLR subgroup without atosiban (*n* = 5) or the low-NLR subgroup with atosiban (*n* = 2) are therefore statistically unreliable and should not inform clinical practice. This interaction *p*-value should not be interpreted as evidence of atosiban efficacy. The comparison is between atosiban-exposed women (*n* = 21) and atosiban-unexposed women (*n* = 5) in the high-NLR stratum, with treatment allocation entirely determined by clinical indication. The observed association is almost certainly confounded by indication and may reflect unmeasured differences between women who were prescribed atosiban and those who were not prescribed atosiban.

Data are presented as mean ± standard deviation. Caution: Table 2 presents observational associations only. The high-NLR/atosiban-naïve cell contains five patients, rendering the estimate statistically unstable. The interaction *p*-value is hypothesis-generating and should not be interpreted as evidence of therapeutic effect. The interaction p-value for NLR stratum × atosiban treatment is 0.026. CL, cervical length.

### Clinical endpoint validation

Cervical shortening rate is a surrogate endpoint; the clinically significant outcomes are gestational age at delivery and preterm birth rates. Among high-NLR women, those who were not receiving atosiban delivered at 30.8 ± 2.2 weeks, with 60.0% delivering at <32 weeks’ gestation and 80.0% at <35 weeks, whereas women who were receiving atosiban treatment delivered at 33.0 ± 1.9 weeks (*p* = 0.048), with reduced preterm birth rates (14.3 and 28.6%, respectively). Neonatal complications decreased from 60.0 to 14.3% in high-NLR women (*p* = 0.002), compared to 3.6% in low-NLR women, with no neonatal deaths in the treated group (Table 3).

**Table 3:** Clinical outcomes and neonatal complications stratified by NLR status and atosiban treatment.

Outcome indicator	High NLR without atosiban (*n* = 5)	High NLR with atosiban (*n* = 21)	Low NLR without atosiban (*n* = 56)	Low NLR with atosiban (*n* = 2)	*p*-value
Pregnancy weeks at the time of delivery					
Average gestational age	30.8 ± 2.2	33.0 ± 1.9	38.5 ± 1.2	38.0 ± 2.8	<0.001
<Delivery at 32 weeks	60.0% (3/5)	14.3% (3/21)	0% (0/56)	0% (0/2)	<0.001
<Delivery at 35 weeks	80.0% (4/5)	28.6% (6/21)	1.8% (1/56)	0% (0/2)	<0.001
Newborn outcome					
Birth weight (g)	1,650 ± 420	1980 ± 380	3,250 ± 450	3,100 ± 560	0.032
Neonatal complications	60.0% (3/5)	14.3% (3/21)	3.6% (2/56)	0% (0/2)	0.002
RDS	40.0% (2/5)	9.5% (2/21)	1.8% (1/56)	0% (0/2)	0.015
Neonatal death	20.0% (1/5)	0% (0/21)	0% (0/56)	0% (0/2)	0.041

Data are presented as mean ± standard deviation or percentage (n/N). NLR, neutrophil-to-lymphocyte ratio.

**Figure 3:**
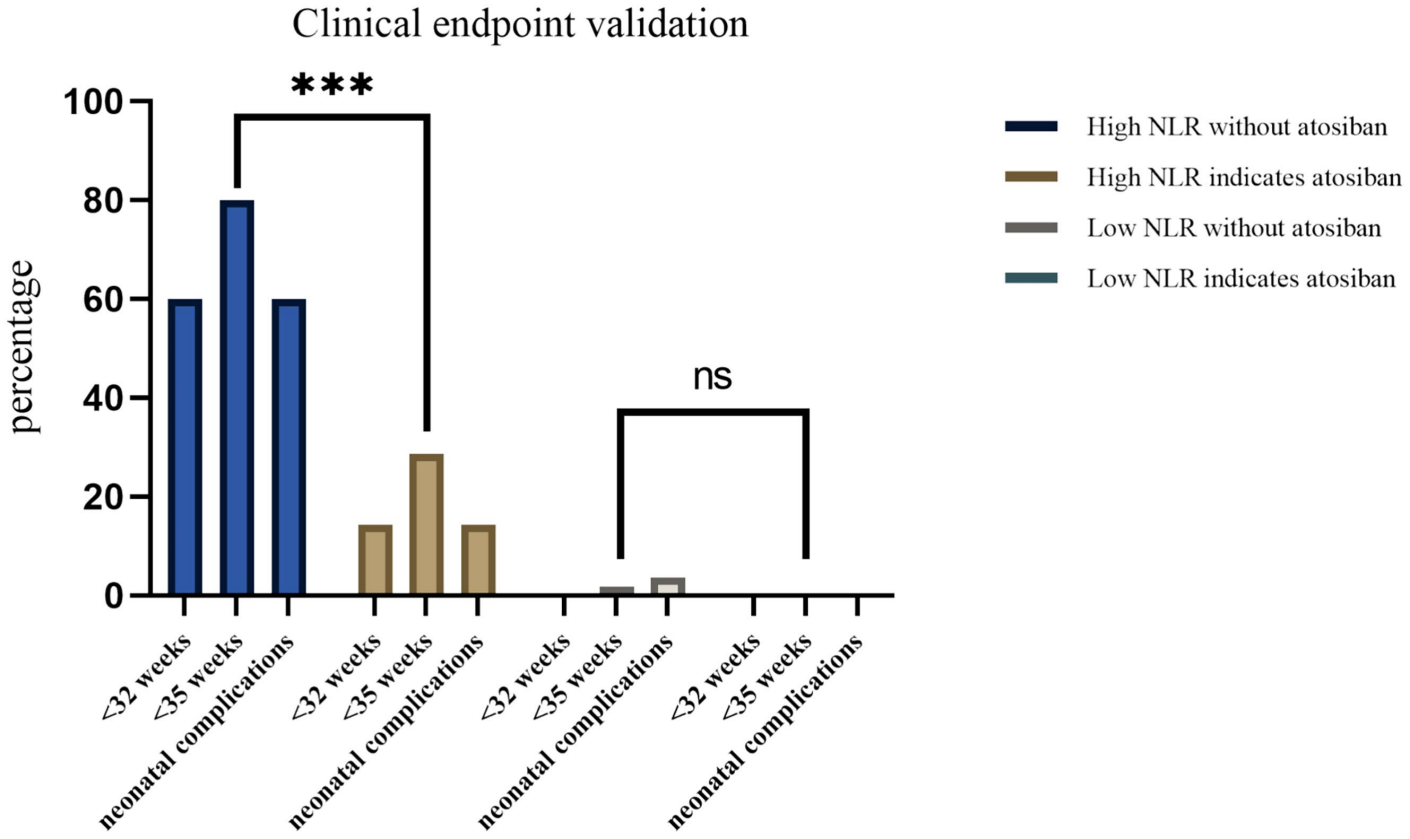
Clinical endpoint validation by NLR status and atosiban treatment. **(A)** Gestational age at delivery; **(B)** Preterm birth rates <32 weeks and <35 weeks; **(C)** Neonatal complication rates; **(D)** Birth weight distribution. *p < 0.05, **p < 0.01, ***p < 0.001 compared to groups.

In contrast, low-NLR women exhibited favorable outcomes regardless of atosiban exposure, delivering at 38.5 ± 1.2 weeks (without atosiban, *n* = 56) and 38.0 ± 2.8 weeks (with atosiban, *n* = 2), with minimal complications (3.6 and 0%, respectively). Notably, atosiban-treated high-NLR women delivered earlier than low-NLR controls (*p* < 0.001) and had higher complication rates (14.3% vs. 3.6%, *p* = 0.015; Figure 3).

## Discussion

### Principal findings in context

Our findings support the hypothesis that a routine pre-operative immunogram—quantified by NLR and CRP—is associated with post-cerclage cervical effacement in this retrospective cohort. Each 1-unit increase in NLR was associated with a 0.09 mm/week increment in CL loss—a magnitude that, accumulated over 10–16 weeks of follow-up—may approach the measurement error of transvaginal ultrasound and corresponds to risk stratification thresholds previously reported in the literature (14). The independence of this association after adjusting for classical anatomical risk factors (baseline CL, prior preterm delivery, BMI, gestational age at cerclage, and uterine contraction status) suggests that systemic inflammation may contribute to cervical remodeling, although causality cannot be established from this observational design. The 0.09 mm/week per-unit NLR association, while statistically significant, is modest compared to transvaginal ultrasound measurement variability (±0.09–0.19 mm/week over 16 weeks). Its primary value lies in risk stratification (identifying a high-risk inflammatory phenotype) rather than in predicting precise millimetric change. We caution against using this coefficient to guide individual patient management.

### Biological plausibility and mechanistic insights

Histological studies have documented polymorphonuclear infiltrates, elevated interleukin-6 (IL-6)/tumor necrosis factor-alpha (TNF-*α*), and increased MMP-9 activity in cervical biopsies weeks before clinical shortening (3, 4). Our data extend these observations to the peripheral circulation: a high NLR may reflect neutrophilic priming, while CRP captures downstream hepatic acute-phase activation. The correlation between NLR and post-operative CRP (r = 0.42) supports a coherent cellular-to-humoral inflammatory axis that may be associated with local collagenolysis via reactive oxygen species and protease release (15). Monocyte enrichment (≥ 0.5 × 10 (9)/L) provided an additive signal, implicating innate mononuclear cells in extracellular matrix remodeling, although this requires confirmation in prospective studies.

### Antibiotics and aseptic inflammation: an observational association

Prolonging intravenous antibiotics ≥ 4 days was associated with lower post-operative CRP but was not associated with attenuated cervical shortening. This observational finding is consistent with the possibility that antimicrobial therapy may have a limited effect on sterile, cytokine-driven remodeling loops (mechanical stretch, prostaglandin, and leukotriene pathways); however, this interpretation is confounded by baseline imbalance and non-random treatment allocation (10, 11). Animal data showing that lipopolysaccharide-independent toll-like receptor-2/4 (TLR-2/4) activation and stretch-induced NF-κB signaling can trigger cervical ripening provide biological plausibility; however, clinical recommendations against routine extended antibiotic prophylaxis cannot be derived from this retrospective analysis. We explicitly refrain from concluding that antibiotics are “ineffective.”

### Atosiban: exploratory observations requiring confirmation

We make no claim that atosiban possesses anti-inflammatory properties *in vivo*, nor that it should be used for cervical remodeling, or that it offers clinical benefit beyond its established tocolytic indication. *Ex vivo* human myometrial models have shown that atosiban can inhibit NF-κB and MAPK activation and reduce COX-2 expression. Our study provides an exploratory clinical observation: In women with pre-existing neutrophilic dominance (NLR ≥ 3.5), atosiban exposure was associated with a 0.15-mm/week decrease in shortening velocity and concurrent lowering of both NLR and CRP, associations that were absent in the low-NLR stratum (12, 13). However, these findings are hypothesis-generating only. The small subgroup sizes (*n* = 5 untreated high-NLR), non-random treatment allocation, and potential for confounding by indication preclude any inference of causal efficacy. Whether atosiban possesses a dual mechanism—uterine quiescence plus cytokine dampening—in cervical tissue remains speculative and requires testing in randomized controlled trials (16, 17).

### Comparison with existing literature

Previous investigations (*n* = 56–78) have focused on post-operative CRP > 10 mg/L as a binary predictor of PTB after cerclage (6, 7). Our study expands the sample to 84 cases, shifts the assessment to the pre-operative window, and incorporates NLR as a continuous variable, achieving reasonable discriminative power (AUC: 0.81). Moreover, by quantifying drug days rather than “use/no-use” categories, we provide exploratory dose–response data that may inform future trial design, although causal inferences are not justified.

### Clinical implications: none at present

We explicitly state that no therapeutic inference regarding atosiban can be drawn from this observational dataset. Laboratory information systems could potentially flag NLR ≥ 3.5 or CRP ≥ 5 mg/L at the time of blood sampling, permitting risk stratification for further studies (8, 9). For women with elevated inflammatory markers, prolonging atosiban beyond the standard 48-h bolus or combining it with progesterone might be hypotheses worth testing in randomized trials, whereas discouraging extended empiric antibiotics based on this observational data would be premature. Future randomized trials should allocate high-NLR patients to standard or intensified regimens, using cervical shortening velocity as a surrogate endpoint, with PTB as the primary clinical outcome. We explicitly discourage any change in clinical practice based on this observational dataset.

### Strengths and limitations

The strengths of this study include complete blood count data, strict sonographic protocol, and drug-duration recording. The limitations are substantial and should be emphasized:

1. Confounding by indication. Atosiban was prescribed for threatened preterm labor; antibiotics were prescribed for suspected infection or prophylaxis. Exposed patients had systematically higher baseline inflammation and obstetric risk. No observational analysis can overcome this bias, and we explicitly preclude causal interpretation of all drug-related findings.
2. Small sample size and extreme subgroup imbalance: With an overall sample size of 84 and cell sizes as small as *n* = 2 and *n* = 5, the study is underpowered for subgroup analyses. Parametric assumptions are violated, and *p*-values from small cells are unreliable.
3. Risk of overfitting: The multivariable model includes 8–10 covariates for 84 patients, approaching one covariate per 8–10 events. Although we performed bootstrap validation, model overfitting remains a concern.
4. Single-center retrospective design: Selection bias, incomplete data, lack of standardized treatment protocols, and absence of external validity limit generalizability.
5. Residual confounding: Despite adjusting for gestational age at cerclage, uterine contraction status, and other clinical factors, unmeasured variables (cervical microbiome, genetic factors, and socioeconomic status) may influence both inflammatory markers and cervical remodeling.
6. Surrogate endpoint uncertainty: Cervical shortening rate is correlated with preterm birth but is not a hard clinical endpoint. The 0.09 mm/week association is of uncertain clinical relevance in terms of ultrasound measurement error.
7. Non-validated biomarker thresholds: The NLR of ≥3.5 and CRP of ≥5 mg/L cutoffs, while supported by the literature, have not been validated in cerclage-specific populations.

## Conclusion

This small, single-center, retrospective cohort study supports the hypothesis that pre-operative NLR and CRP are associated with cervical shortening after cerclage. All drug-related observations are confounded by indication and based on statistically unstable subgroup estimates. No causal inference or clinical recommendation can be made based on these findings. The only valid conclusion is that these routine biomarkers warrant prospective evaluation in larger, randomized studies with preterm birth as the primary endpoint.

## Data Availability

The original contributions presented in the study are included in the article/supplementary material, further inquiries can be directed to the corresponding authors.
